# 625 Implementing Burn Padlet to Improve Interprofessional Collaboration Within the BTSD Interdisciplinary Team

**DOI:** 10.1093/jbcr/iraf019.254

**Published:** 2025-04-01

**Authors:** Rhoda Palacio, Olivia Iacobelli

**Affiliations:** University of Rochester Medical Center; University of Rochester Medical Center

## Abstract

**Introduction:**

The interprofessional collaboration within the interdisciplinary team is the key concept necessary to provide safe high-quality care for burn patients. Nurses must be knowledgeable and confident in caring for burn patients, therefore resources must be available and easily accessible. The development and implementation of technology-based collaborative learning tools such as Burn Padlet is a quality improvement project developed to enhance providers’ and nurses’ understanding of workflow, standards of care, and the latest evidence-based clinical practice guidelines.

**Methods:**

A pre-implementation qualitative survey was done to assess baseline knowledge and understanding of burn-related information, and the level of communication between teams. The results demonstrated a need for burn resources readily available within the service to promote standardization of care to all burn patients. The Burn Padlet is a comprehensive resource tool that was developed utilizing the evidence-based practice guidelines and standards of care from each interdisciplinary team’s department. The nursing staff’s input and the Burn Nurse Competency education were also incorporated into this Padlet to further improve the competency level of all nursing staff.

**Results:**

The post-implementation results were obtained 6 months after the quality improvement project was initiated into practice. The findings suggest an increase in burn nurses’ knowledge of burn-related information, providers’ workflow, and communication between nursing staff and providers. Nursing staff stated that they have a better understanding of burn protocols, guidelines, and procedures. Staff also reported that the tool is useful in preventing communication barriers between nurses and providers. The goal of this project is to continue to further develop this tool to reflect the most current evidence-based practices in the burn patient population.

**Conclusions:**

This quality improvement project has met the goals for patient care needs. The intended outcomes of this project include but are not limited to optimizing burn resuscitation, nutritional needs, mobility, wound care, post-op care, infection prevention, pain management, and the effective utilization of resources available. The Burn Trauma ICU and Med/Surg units also utilize the Padlet as a resource to meet the needs of their burn patients.

**Applicability of Research to Practice:**

Research evidence has shown interprofessional collaboration has proven to reduce medical errors, promote safety culture, and improve patient outcomes. Nurses have a pivotal role in influencing innovation, collaboration, leading across the continuum of care, piloting new ideas, and expanding the accessibility and use of technology for hospital operations, and research. Therefore, the successful implementation of an innovative tool to improve interdisciplinary communication is an essential component to achieving patient satisfaction and quality patient care.

**Funding for the Study:**

N/A

